# Prevalence and Risk of Homelessness Among US Veterans

**Published:** 2012-01-26

**Authors:** Jamison Fargo, Stephen Metraux, Thomas Byrne, Ellen Munley, Ann Elizabeth Montgomery, Harlan Jones, George Sheldon, Vincent Kane, Dennis Culhane

**Affiliations:** National Center on Homelessness Among Veterans and Utah State University Department of Psychology; National Center on Homelessness Among Veterans and University of the Sciences, Philadelphia, Pennsylvania; National Center on Homelessness Among Veterans and University of Pennsylvania, Philadelphia, Pennsylvania; National Center on Homelessness Among Veterans and The City University of New York, Philadelphia, Pennsylvania; National Center on Homelessness Among Veterans and University of Pennsylvania, Philadelphia, Pennsylvania; National Center on Homelessness Among Veterans and University of Pennsylvania, Philadelphia, Pennsylvania; US Department of Veterans Affairs, Washington, DC; National Center on Homelessness Among Veterans, Philadelphia, Pennsylvania; National Center on Homelessness Among Veterans and University of Pennsylvania, Philadelphia, Pennsylvania

## Abstract

**Introduction:**

Understanding the prevalence of and risk for homelessness among veterans is prerequisite to preventing and ending homelessness among this population. Homeless veterans are at higher risk for chronic disease; understanding the dynamics of homelessness among veterans can contribute to our understanding of their health needs.

**Methods:**

We obtained data on demographic characteristics and veteran status for 130,554 homeless people from 7 jurisdictions that provide homelessness services, and for the population living in poverty and the general population from the American Community Survey for those same jurisdictions. We calculated prevalence of veterans in the homeless, poverty, and general populations, and risk ratios (RR) for veteran status in these populations. Risk for homelessness, as a function of demographic characteristics and veteran status, was estimated by using multivariate regression models.

**Results:**

Veterans were overrepresented in the homeless population, compared with both the general and poverty populations, among both men (RR, 1.3 and 2.1, respectively) and women (RR, 2.1 and 3.0, respectively). Veteran status and black race significantly increased the risk for homelessness for both men and women. Men in the 45- to 54-year-old age group and women in the 18- to 29-year-old age group were at higher risk compared with other ages.

**Conclusion:**

Our findings confirm previous research associating veteran status with higher risk for homelessness and imply that there will be specific health needs among the aging homeless population. This study is a basis for understanding variation in rates of, and risks for, homelessness in general population groups, and inclusion of health data from US Department of Veterans Affairs records can extend these results to identifying links between homelessness and health risks.

## Introduction

Veterans are overrepresented among the homeless in the United States and are at greater risk than nonveterans of becoming homeless (1-10). Homelessness is associated with chronic health conditions, either causing or preceding such conditions, becoming a consequence of such conditions, or complicating the treatment and care of such conditions (11-14). Furthermore, among the 136,000 homeless veterans in 2009, 53% had a chronic health condition (15). Understanding the epidemiology of homelessness and the specific factors associated with increased risk of becoming homeless is prerequisite to both reducing homelessness and more effectively addressing the health needs of this population.

The objective of this study was to provide a more detailed assessment of risk for homelessness among veterans than has been previously reported, in comparison with the nonveteran population and after controlling for various demographic characteristics. Specifically, we sought to answer 2 research questions: 1) Is veteran status associated with an increased risk of homelessness? and 2) Does risk of homelessness among veterans vary as a function of age, race, and sex?

## Methods

### Study design

Homeless Management Information Systems (HMIS) and American Community Survey (ACS) data from 7 jurisdictions provided a basis for estimating the prevalence of veterans in the homeless, poverty, and overall populations; calculating risk ratios for veteran status in the homeless population compared with veteran status in the poverty and overall populations; determining if veteran status is associated with an increased risk of homelessness; and identifying whether risk of homelessness among veterans varies as a function of age, race, or sex.

### Data collection

Data for this study came from the 2008 HMIS and the 2006-2008 ACS*. *Service providers use HMIS to record data on client characteristics and use of services in homeless populations across a local area known as a continuum of care (CoC). A CoC is a planning entity established by the US Department of Housing and Urban Development (HUD) for a geographic unit, which can range in size from a large city to multiple rural counties. In a CoC, stakeholders and service providers coordinate resources and provide services (eg, shelter, housing, food) to address homelessness (16). The more than 400 CoCs throughout the United States are each mandated by HUD to maintain an HMIS that collects data on the local service-using homeless population. The data fields collected include identifying information, veteran status, demographics, the presence of disabling conditions, and dates of program entry and exit.

A convenience sample of 11 urban CoCs from geographic regions throughout the country initially provided HMIS data for this study. These HMIS datasets consisted of unduplicated, de-identified, individual records for adults who used emergency shelter or transitional housing within their CoC during 2008. HMIS data from 7 of these 11 jurisdictions were usable and sufficiently complete (<10% missing): New York, New York; San Jose/Santa Clara County, California; Columbus/Franklin County, Ohio; Denver, Colorado (Denver, Adams, Arapahoe, Boulder, Broomfield, Douglas, and Jefferson counties); Tampa/Hillsborough County, Florida; Phoenix/Maricopa County, Arizona; and Lansing/Ingham County, Michigan.

We estimated data missing because of nonresponse to an item in the dataset (17) (ie, missing 1 or more data elements) from these CoCs by using single imputation techniques and SOLAS version 3.2 (Statistical Solutions, Saugus, Massachusetts). Some users of homeless services were not included in HMIS data because of the service providers' lack of participation; this unit nonresponse was addressed by applying a variation of the extrapolation procedures used in the Annual Homeless Assessment Report (AHAR) to estimate additional homeless people (veterans and nonveterans) who used homeless services but were not recorded doing so (18). Extrapolation procedures produce reliable estimates when more than half of providers in a CoC participate in HMIS (ie, <50% data missing because of unit nonresponse); all CoCs included in this study were well above this threshold. Extrapolation increased our homeless sample by 20,964 people (2,455 veterans, 18,509 nonveterans), a 16% increase over the original sample.

To compute rates of homelessness, we used ACS data to estimate the total veteran and nonveteran populations in each CoC. The ACS is an annual survey administered by the US Census Bureau that collects social, economic, and demographic information from samples of housing units in all counties in the United States (19). We selected 3-year estimates (2006-2008) for this study because they are based on a larger sample size than the 1-year estimates and offer better precision, especially in examining smaller populations such as veterans, and smaller geographic areas. When CoC boundaries varied from the geographic areas for which ACS estimates are publicly available, the US Census Bureau provided customized ACS estimates for these CoCs. For each geographic area, we aggregated ACS data by age, sex, race, veteran status, and poverty status.

This study received approval as an exempt study from institutional review boards at the University of Pennsylvania and the US Department of Veterans Affairs (VA).

### Outcomes

Homelessness status was our outcome of interest. Data collected through HMIS for the homeless population included age (18-29, 30-44, 45-54, 55-64, >65 y), race (black, nonblack), sex, and veteran status (veteran, nonveteran). ACS variables included in this study were age, race, sex, and veteran status in categories consistent with HMIS data. In addition, ACS data were stratified by poverty status, that is, whether household income was below the federal poverty threshold. All people in the HMIS database were considered as living in poverty on the basis of their homeless status. ACS, which collects data from group quarters in addition to private housing units, included both homeless and housed members of the population but did not differentiate the population on this basis. Veteran status is defined as having served in the US military and is based on self-report in both HMIS and ACS data.

### Data analysis

Two phases of analysis used pooled data from the 7 CoCs. All analyses were weighted by CoC size, were conducted separately for men and women as well as for the total population and for the population living in poverty (from the ACS), and were conducted using the R language and environment for statistical computing, version 2.13 (R Foundation for Statistical Computing, Vienna, Austria) (20).

In the first phase, we estimated the prevalence of veterans in the homeless, poverty, and overall populations and calculated corresponding risk ratios (RR). This process provided a simple measure of whether veterans were overrepresented in the homeless population. We computed prevalence and risk ratios for each age, race, and sex subgroup. Risk ratios for men and women were age- and race-adjusted.

In the second phase, we conducted binomial generalized estimating equation (GEE) analyses in which homeless status was the outcome, and age, race, and veteran status were potential predictors. Because we were modeling frequencies, the outcome was a ratio of homeless (from HMIS data) to total (general or poverty population from ACS data) people for each subpopulation, as defined by the frequency within each subgroup, weighted by that same frequency (21). GEE modeling adjusted for dependence because of clustering within individual CoCs. The phase 2 analysis consisted of main-effects-only multivariate models. Three interaction effects were selected *a priori* and tested but were later discarded because they were found to be nonsignificant: veteran status by 1) age, 2) race, and 3) age by race.

## Results

### Phase 1 results

An estimated 130,554 adults received homelessness services in the 7 CoCs in this study; 10,726 of these adults (8.2%) reported veteran status (Table 1). This rate was higher than the veteran rate among the ACS poverty (n = 63,655, 3.34%) and ACS general (n = 1,023,515, 6.96%) populations. Compared with nonveterans, veterans in each population (HMIS, ACS poverty, ACS general) were disproportionately male and in the older age categories.

Veterans were overrepresented in the homeless population for both sexes (Table 2). For men, 13.6% of the homeless adults were veterans, whereas for women 1.8% of homeless adults were veterans. These rates yielded age- and race-adjusted RRs of 2.1 (men) and 3.0 (women) compared with the population living in poverty, and 1.3 (men) and 2.1 (women) compared with the general population. RRs for demographic subgroups were generally consistent with the overall RRs.

The age- and race-adjusted RRs for homelessness among both men and women were higher for veterans than for nonveterans in both the poverty (RR, 2.2 for men and 3.0 for women) and general populations (RR, 1.4 for men and 2.3 for women) (Table 3). Rates of homelessness were consistently higher in veteran populations than in nonveteran populations, and among both veterans and nonveterans, black adults, especially in the younger age groups, had higher rates of homelessness.

### Phase 2 results

Veteran status, older age, and black race were significantly and independently associated with risk of homelessness among both men and women. Similarly, the patterns of results found in the general population were consistent with those found in the poverty population; however, in the latter, veteran status was associated with a greater risk for homelessness.

For the veteran indicator, male veterans were almost 50% as likely (adjusted odds ratio [AOR], 1.47; 95% confidence interval [CI], 1.19-1.81) and female veterans were almost twice as likely (AOR, 1.97; 95% CI, 1.25-3.12) to be homeless as nonveterans in the general population. Among the population in poverty, male veterans were more than twice as likely (AOR, 2.20; 95% CI, 1.96-2.48) and female veterans were more than 3 times as likely (AOR, 3.33; 95% CI, 2.17-5.13) to be homeless as nonveterans.

Among the control variables, increased age was significantly associated with homelessness, but its effect differed between sexes. Among men, risk for homelessness generally increased as a function of age up to the 45- to 54-year-old age range, but declined thereafter. This was so among both veterans and nonveterans and in both the general and poverty populations. Men in the 45- to 54-year-old age group appeared to be at the highest risk of homelessness, nearly twice as likely (AOR, 1.85; 95% CI, 1.18-1.93) in the general population and 3 times as likely (AOR, 2.65; 95% CI, 1.41-4.97) in the poverty population as their 18- to 29-year-old counterparts to be homeless. Male veterans in the 45- to 54-year-old age group made up 41% of the homeless veterans. Risk for homelessness among women declined with age at an increasing rate in both the general and poverty populations, so that older women were at the lowest risk for homelessness, compared with the youngest group.

Finally, black race was also a significant predictor of homelessness among all subgroups. In the general population, the risk associated with black race increased more than 5-fold for both men and women (AOR, 5.49; 95% CI, 4.25-7.09 for men and AOR, 5.45; 95% CI, 4.23-7.01 for women). This risk was lower in the poverty population but remained high; the AOR for men was 2.18 (95% CI, 1.95-2.45) and for women was 3.32 (95% CI, 2.16-5.11).

## Discussion

The findings in this report support those of earlier studies that showed veterans to be overrepresented in the homeless population and reach beyond by showing veteran status to be associated with increased risk for homelessness after controlling for race, sex, and age. The magnitude of this association became greater after controlling for poverty; veteran status was associated with more than a 2-fold increase for men and a 3-fold increase for women in the odds of becoming homeless.

For male veterans, those in the 45- to 54-year-old age group made up 41% of the homeless veterans and also had the highest risk for becoming homeless. This finding is consistent with other research (2) that identified a cohort effect in this age group of veterans. This cohort, whose key characteristic was service during the initial years of the All Volunteer Force, instituted in 1973, has continuously been the veteran age group at highest risk for homelessness as these veterans have aged over the last 2 decades. Similarly, members of the general population who are now aged 45 to 54 have continuously been at highest risk for homelessness (21-23).

Veterans make up a discrete subgroup in this general age cohort, in terms of both the increased risk for homelessness associated with their veteran status and their access to health care and homeless services through the VA. The susceptibility of homeless people to chronic disease and disability increases as they age, and the veterans among them will increasingly turn to the VA for health care. Given their lack of housing and heightened susceptibility to chronic health problems, homeless veterans will likely contribute disproportionately to the increased demand for long-term care through the VA (24). But beyond that, the changing health and need for housing support services of an aging homeless population are poorly understood. As the VA responds to an aging veteran population through increased reliance on community-based care to treat chronic illness (25), those with the most tenuous ties to the community will be the ones who present the most pressing challenges.

Among women, particularly black women, the youngest age groups were at highest risk for homelessness. This finding is consistent with media accounts that women who served in more recent conflicts such as those in Iraq and Afghanistan are more likely than older female veterans to be homeless (26). This finding is also consistent with other research indicating that among women in general, the period of highest vulnerability for homelessness is during the time period when they are heading families with young children (27). Because younger cohorts are most at risk, female veterans stand to benefit more from existing homelessness-prevention efforts tied to reentering civilian life, which focus on housing needs, than from efforts that combine housing with health care services.

Veterans who are living in poverty are more vulnerable to homelessness, an effect that is magnified by black race. For example, for the youngest age group living in poverty, more than 50% of black male veterans and more than 30% of black female veterans were homeless (compared with only 7% for nonblack males and 12% for nonblack females), according to HMIS data. These alarmingly high rates suggest that homelessness-prevention activities—including tenant/landlord mediation or short-term rent and utility payments—among veterans may be particularly effective because they can target a finite poverty population and can further refine this effort by focusing on black veterans. Our findings highlight the usefulness of these data for such targeting, but future investigations of risk factors must go beyond the simple focus on race and poverty status. The addition of health-related data to the datasets used here could make specific links between health conditions and risk for homelessness. The VA is currently building a registry of veterans using homelessness services that can be linked to VA health care records, which promises such assessments of health-related risks for homelessness and for which this study could be a prototype.

Although the 7 CoCs included in our study represented approximately 10% of the US homeless population, they are a convenience sample of urban jurisdictions, which limits our study's comparability to other studies. This difference likely contributed to the divergence in a key finding between this study and the Veteran Supplement to the Annual Homelessness Assessment Report (15). Whereas this study demonstrated that male veterans were overrepresented among the homeless population (RR, 1.3), the Vet-AHAR found them to be underrepresented (RR, 0.7). This disparity is explained in part by the differences in geographic areas, as the Vet-AHAR was a nationally representative estimate. Further explanation for this difference in findings is the Vet-AHAR's inability to adjust its risk assessments by age and race.

Another limitation of our study is that the veteran status was based on self-report and likely included people who reported veteran status but may have been ineligible for VA services. Conversely, we may have included people eligible for VA services who did not acknowledge veteran status. The HMIS data are also limited in their universally available data fields, and a more comprehensive range of data fields would go further toward understanding and eliminating homelessness.

In conclusion, this study offers evidence that supports and expands on prior findings that veterans, particularly older veterans, are vulnerable to homelessness. As more and richer data on veteran homelessness, and homelessness in general, become available through HMIS and other administrative sources, future research should be able to increasingly relate health data to the demographic characteristics included in this study.

## Figures and Tables

**Table 1:** Demographic and Geographic Characteristics of Homeless People in Selected US Metropolitan Areas^a^

Characteristic	HMIS Homeless Population^b^(n = 130,554)		ACS Poverty Population^c^(n = 1,905,110)		ACS General Population^d^(n = 14,708,440)	

	Veteran, % (n = 10,726)	Nonveteran, % (n = 119,828)	Veteran, % (n = 63,655)	Nonveteran, % (n = 1,841,455)	Veteran, % (n = 1,023,515)	Nonveteran, % (n =13,684,925)
**Age, y**						
<29	6.8	32.4	6.2	33.6	4.3	24.9
30-44	24.0	38.5	14.2	28.1	15.3	31.3
45-54	40.8	21.0	20.0	14.5	15.0	18.5
55-64	23.3	6.7	25.5	10.2	25.4	12.5
≥65	5.1	1.4	34.1	13.7	40.1	12.9
**Sex**						
Female	10.2	48.9	9.8	60.2	6.8	54.8
Male	89.8	51.1	90.2	39.8	93.2	45.2
**Race**						
Black	46.0	46.9	21.2	19.4	11.4	13.9
Nonblack	54.0	53.1	79.8	80.6	88.6	86.1
**CoC metropolitan area**						
Columbus, Ohio	6.4	4.4	7.2	6.1	7.5	5.6
Denver, Colorado	7.6	3.3	16.3	10.6	19.5	13.5
Lansing, Michigan	2.4	1.7	2.0	2.0	1.6	1.5
New York City	36.5	62.2	35.4	54.8	24.5	45.7
Phoenix, Arizona	20.2	12.3	24.9	16.1	29.2	18.7
San Jose, California	17.5	12.0	5.9	5.3	7.6	9.2
Tampa, Florida	9.3	4.1	8.3	5.1	10.1	5.8

Abbreviations: HMIS, Homeless Management Information System; ACS, American Community Survey; CoC, Continuum of Care.

a Source: CoC data are collected for geographic units established by the US Department of Housing and Urban Development to track resource use for homeless populations.

b People within a CoC who used homelessness services, according to HMIS 2008.

c Adults identified by the ACS 2006-2008 whose incomes fell below the federal poverty threshold.

d ACS, 2006-2008.

**Table 2:** Prevalence and Risk of Veteran Status in Homeless, Poverty, and Overall Populations in 7 US Metropolitan Areas^a^

**Characteristic**		Veterans in Homeless Population^b^, % (n = 10,726)		Veterans in Poverty Population^c^, % (n = 63,655)		RR^d^		Veterans in General Population^e^, % (n = 1,023,515)		RR^f^	

**Age, y**	**Race**	M	F	M	F	M	F	M	F	M	F
18-29	Black	3.8	1.0	0.9	0.4	4.2	2.2	1.9	0.6	2.0	1.7
	Nonblack	2.7	1.0	1.3	0.3	2.2	3.1	2.1	0.5	1.3	2.0
30-44	Black	8.2	3.2	5.9	1.3	1.4	2.5	7.3	1.6	1.1	1.9
	Nonblack	7.6	1.3	3.5	0.4	2.1	2.9	5.9	0.8	1.3	1.6
45-54	Black	21.0	2.7	14.7	1.0	1.4	2.6	14.7	1.7	1.4	1.6
	Nonblack	19.6	3.1	9.2	1.1	2.1	2.9	9.8	1.2	2.0	2.5
55-64	Black	31.9	1.8	20.8	0.8	1.5	2.3	23.0	0.9	1.4	1.9
	Nonblack	30.6	3.1	19.0	0.6	1.6	4.9	27.6	1.0	1.1	3.1
≥65	Black	32.3	1.4	26.7	0.5	1.2	2.9	33.2	0.6	1.0	2.6
	Nonblack	33.7	2.4	21.9	0.9	1.5	2.8	45.4	1.1	0.7	2.1
All ages^g^	Black	13.7	2.0	9.4	0.8	2.4	2.5	11.8	1.1	1.4	1.9
	Nonblack	13.4	1.6	7.4	0.6	2.0	3.1	13.6	0.9	1.3	2.1
All ages^h^	All races	13.6	1.8	7.8	0.6	2.1	3.0	13.4	0.9	1.3	2.1

Abbreviations: M, male; F, female; RR, risk ratio.

a Continuum of Care (CoC) data are collected for geographic units established by the US Department of Housing and Urban Development to track resource use for homeless populations. The 7 CoC metropolitan areas included in this analysis are Columbus, Ohio; Denver, Colorado; Lansing, Michigan; New York City; Phoenix, Arizona; San Jose, California; and Tampa, Florida.

b People within a CoC who used homelessness services, according to Homeless Management Information System 2008.

c Adults whose incomes fell below the federal poverty threshold, according to the American Community Survey (ACS) 2006-2008.

d Prevalence of veterans in homeless population divided by prevalence of veterans in poverty population.

e ACS 2006-2008.

f Prevalence of veterans in homeless population divided by prevalence of adults in general population.

g Risk ratios are age-adjusted.

h Risk ratios are both age- and race-adjusted.

**Table 3:** Prevalence and Risk of Homelessness^a^ Among Veterans and Nonveterans in Poverty and General Populations in 7 US Metropolitan Areas^b^

**Characteristic**		Homelessness Among Veterans in Poverty Population^c^, %		Homelessness Among Nonveterans in Poverty Population^c^, %		RR^d^		Homelessness Among Veterans in General Population^e^, %		Homelessness Among Nonveterans in General Population^e^, %		RR^f^	
**Age, y**	**Race**	M	F	M	F	M	F	M	F	M	F	M	F
18-29	Black	52.8	36.3	11.8	15.7	4.5	2.3	5.4	7.9	2.6	4.6	2.1	1.7
	Nonblack	7.3	11.9	3.3	3.9	2.2	3.1	0.7	1.6	0.5	0.8	1.4	2.1
30-44	Black	33.8	35.4	23.7	13.8	1.4	2.6	4.7	6.3	4.1	3.2	1.1	2.0
	Nonblack	17.2	12.1	7.7	4.4	2.2	2.8	1.0	0.9	0.7	0.6	1.3	1.5
45-54	Black	38.0	29.1	24.6	10.7	1.5	2.7	7.3	3.2	4.8	2.0	1.5	1.6
	Nonblack	21.0	12.3	8.7	4.1	2.4	3.0	1.9	1.1	0.9	0.4	2.2	2.7
55-64	Black	24.2	9.1	13.6	3.7	1.8	2.4	3.8	1.4	2.4	0.7	1.6	2.1
	Nonblack	10.5	9.3	5.6	1.8	1.9	5.2	0.6	0.6	0.6	0.2	1.1	3.3
≥65	Black	4.8	1.7	3.6	0.6	1.3	2.8	0.6	0.4	0.6	0.1	1.0	3.2
	Nonblack	2.1	0.8	1.2	0.3	1.8	2.9	0.1	0.1	0.1	0.0	0.7	2.3
All ages^g^	Black	26.8	29.7	17.7	11.6	2.5	2.5	4.0	4.9	3.4	2.7	1.5	2.1
	Nonblack	10.6	9.2	5.5	3.3	2.2	3.2	0.6	0.8	0.7	0.5	1.4	2.3
All ages^h^	All races	14.6	15.0	7.9	5.1	2.2	3.0	1.0	1.6	1.0	0.8	1.4	2.3

Abbreviations: M, male; F, female; RR, risk ratio.

a People within a Continuum of Care (CoC) who used homelessness services, according to Homeless Management Information System 2008.

b CoC data are collected for geographic units established by the US Department of Housing and Urban Development to track resource use for homeless populations. The 7 CoC metropolitan areas included in this analysis are Columbus, Ohio; Denver, Colorado; Lansing, Michigan; New York, New York; Phoenix, Arizona; San Jose, California; and Tampa, Florida.

c People whose incomes fell below the federal poverty threshold, according to the American Community Survey (ACS) 2006-2008.

d Prevalence of homelessness among veterans divided by prevalence of homelessness among nonveterans in poverty population.

e ACS 2006-2008.

f Prevalence of homelessness among veterans divided by prevalence of homelessness among nonveterans in the general population.

g Risk ratios are age-adjusted.

h Risk ratios are both age- and race-adjusted.
